# The Role of Gut Microbiome in Hepatocellular Carcinoma: A Systematic Review

**DOI:** 10.7759/cureus.43862

**Published:** 2023-08-21

**Authors:** Yash Trivedi, Zoryana Bolgarina, Heet N Desai, Mithum Senaratne, Shivling S Swami, Soe Lwin Aye, Lubna Mohammed

**Affiliations:** 1 Internal Medicine, California Institute of Behavioral Neurosciences & Psychology, Fairfield, USA; 2 Obstetrics and Gynecology, California Institute of Behavioral Neurosciences & Psychology, Fairfield, USA

**Keywords:** gastrointestinal microbiome, gut flora, gut microbiome, hepatocellular carcinoma (hcc), liver cancer

## Abstract

Gut microbiome dysbiosis is common in patients with chronic liver diseases such as hepatocellular carcinoma (HCC) and plays an essential role in developing, diagnosing, and treating HCC. The purpose of this systematic review, which was carried out following the Preferred Reporting Items for Systematic Review and Meta-analyses 2020 guidelines, is to determine the role of the gut microbiome in the pathogenesis, diagnosis, and treatment of HCC. We collected and reviewed articles, including clinical trials, literature reviews, case-control studies, cross-sectional studies, cohort studies, systematic reviews, and meta-analyses, published between May 30, 2013, and May 30, 2023. The databases used to collect these articles included PubMed, Cochrane Library, Google Scholar, and ScienceDirect. After applying appropriate filters, a total of 2,969 studies were identified. They were further screened and subjected to quality assessment tools which finally yielded 17 studies included in this systematic review. This systematic review provides information regarding the gut-liver axis and the relationship between gut microbiome dysbiosis and HCC.

## Introduction and background

Hepatocellular carcinoma (HCC) is the most common primary liver cancer whose common risk factors include chronic hepatitis B (HBV) and hepatitis C virus (HCV) infection, alcoholic liver disease, and non-alcoholic liver disease. These risk factors lead to liver injury and fibrinogenesis, resulting in cirrhosis and HCC [1]. Liver cancer is the sixth most common malignancy and the fourth most common cause of cancer-related death. Worldwide, HCC is the seventh most common cancer in women and fifth in men [2]. According to an estimate, between 2015 and 2030, 20 million people will die from HBV-related hepatitis, cirrhosis, and HCC, and almost five million will die from HCC itself [3].

The human microbiome consists of bacteria and viruses and can alter our physiology as they play a role in metabolism and immunity. The gut microbiome, also known as gut flora, plays an important role in digestion and nutrition, and recent studies have shown how the gut microbiome and humans have developed a beneficial relationship to both [4]. The gut microbiome is mainly composed of strict anaerobes and, to a lesser extent, facultative anaerobes and aerobes. Out of more than 50 bacterial phyla detected in the human gut, Bacteroidetes and Firmicutes are the most common [5].

Recent studies have shown an association between the gut microbiome and inflammatory diseases, metabolic diseases, and tumors. B. Hoefert, in 1921, found a relationship between the gut microbiome and chronic liver disease, supported by recent studies that show an altered gut microbiome in chronic liver disease and liver cancer patients [3,6-8]. Recently, it has been found that the gut microbiome regulates the activity of peroxisome proliferator-activated receptors, which, in turn, affects intestinal inflammation and gut mucosal permeability [9]. In cirrhotic patients, there are increased levels of lipopolysaccharide (LPS) which is a metabolite produced by the gut microbiome, possibly due to increased gut permeability resulting in increased bacterial translocation, which then activates the nuclear factor kappa-light-chain-enhancer of activated B cells pathway causing the release of proinflammatory cytokines leading to liver inflammation [10,11]. Studies have also shown that the gut microbiome uses bile acids to regulate the activity of natural killer T (NKT) cells in the liver, which have anti-tumor activity by altering various cytokines [12-14]. A study demonstrated that interleukin-25 (IL-25) promotes HCC progression by activating the alternate pathway of macrophages, which, in turn, induces epithelial-mesenchymal translocation [15]. Recent studies have shown that innate and acquired immunity can either promote or inhibit tumorigenesis, which is explained by the fact that even though they are responsible for immune surveillance, they can cause chronic inflammation, which can, in turn, cause carcinogenesis [16].

In recent years, much research has been done on the gut microbiome and its role in chronic liver diseases and HCC. Although gut microbiome dysbiosis is considered an environmental factor that is also affected by host factors such as genetic background, health status, and lifestyle and is also found to play a role in many diseases, its specific contribution remains vague. Different microbes have been associated with different diseases, but knowledge regarding the causal direction of association remains minuscule. Moreover, most of the studies are done on animals and fecal samples of patients, but most of the gut-liver axis alterations occur in the small intestine. This systematic review aims to discuss the role of gut microbiota in the pathogenesis, diagnosis, and treatment of HCC.

## Review

Methodology

This systematic review was carried out in accordance with the Preferred Reporting Items for Systematic Reviews and Meta-Analyses (PRISMA) 2020 recommendations [17].

Search Strategy

A thorough literature search was done on databases such as PubMed, Cochrane Library, Google Scholar, and ScienceDirect. The search was done using keywords such as “hepatic cancer,” “hepatocellular carcinoma,” “hepatic neoplasm,” “liver cancer,” “hepatocellular cancer,” “gastrointestinal microbiome,” and “gut microbiome” and combining them using Boolean operators such as “AND” and “OR.” A Medical Subject Headings strategy was used to narrow the published articles. Table 1 summarizes the databases screened for the collection of articles and the search strategy used for the same.

**Table 1:** Databases used for collecting articles (along with search strategies and appropriate filters).

Type of database	Keywords	Search strategy	Filters used	Number of records identified
PubMed	Hepatic cancer, hepatocellular carcinoma, hepatic neoplasm, liver cancer, hepatocellular cancer, gastrointestinal microbiome	hepatic neoplasm OR hepatic cancer OR hepatocellular cancer OR liver neoplasm OR liver cancer OR (“Liver Neoplasms/diet therapy”[Majr] OR “Liver Neoplasms/etiology”[Majr] OR “Liver Neoplasms/genetics”[Majr] OR “Liver Neoplasms/immunology”[Majr] OR “Liver Neoplasms/metabolism”[Majr] OR “Liver Neoplasms/microbiology”[Majr] OR “Liver Neoplasms/pathology”[Majr] OR “Liver Neoplasms/physiopathology”[Majr] OR “Liver Neoplasms/prevention and control”[Majr] ) AND “Gastrointestinal Microbiome”[Majr]	Free full text, last 10 years, English	327
Cochrane Library	Gut microbiome, liver cancer	Gut microbiome AND liver cancer	Published between 2013 and2023	21
Google Scholar	Gut microbiome, hepatocellular carcinoma, liver cancer	Gut microbiome AND hepatocellular carcinoma OR liver cancer	Published between 2013 and 2023	65
ScienceDirect	Gut microbiome, liver cancer	Gut microbiome AND liver cancer	Published between 2013 and 2023, open access and open archive	2,556

Inclusion and Exclusion Criteria

Only free full-text articles written in the English language and published within the last 10 years between May 30, 2013, and May 30, 2023, were taken into consideration. All study designs were taken into consideration for this systematic review. Gray literature was not included in this review.

Results

**Figure 1:**
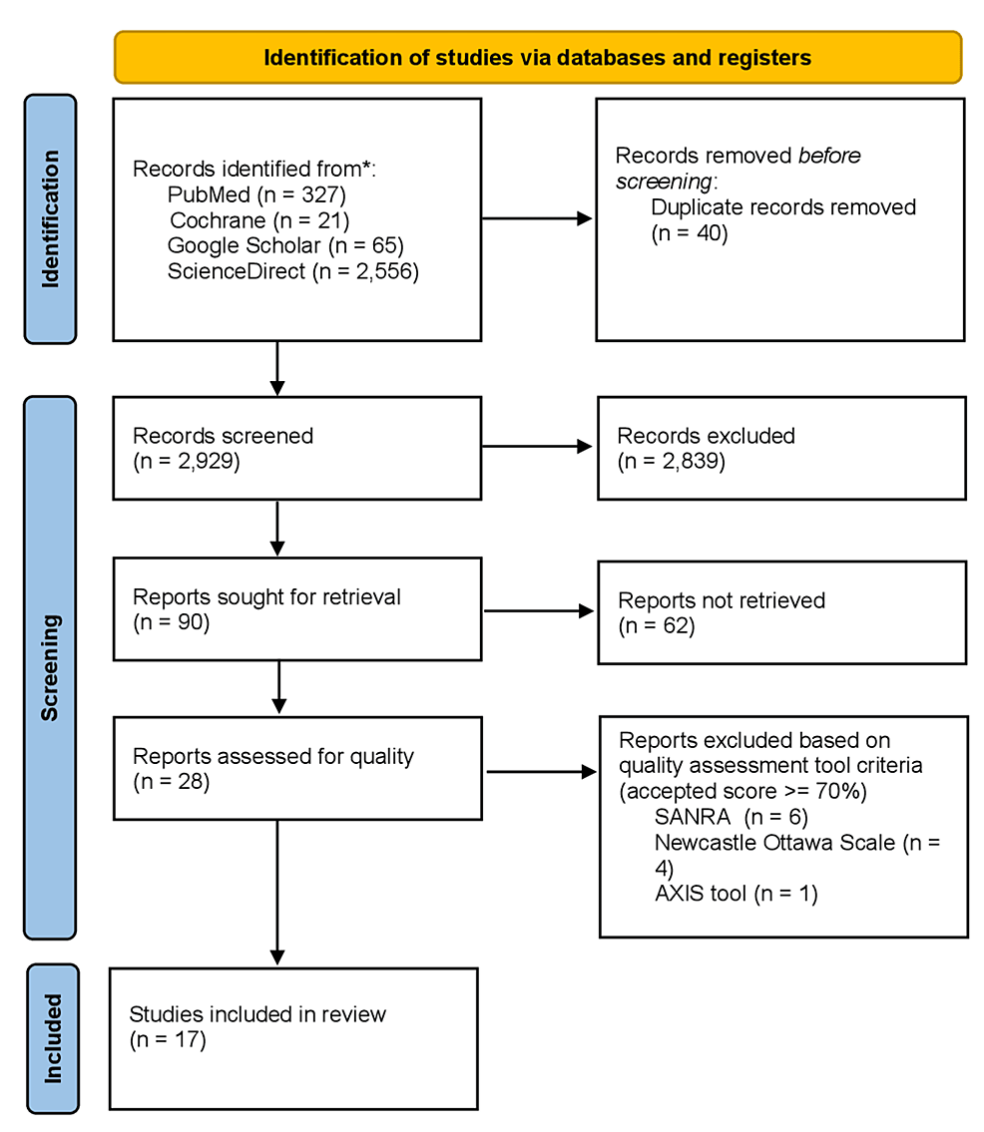
PRISMA chart illustrating the screening process and quality assessment of the articles. SANRA: Scale for the Quality Assessment of Narrative Review Articles; PRISMA: Preferred Reporting Items for Systematic Reviews and Meta-Analyses; AXIS tool: Appraisal Tool for Cross-Sectional Studies

Using a search strategy and appropriate filters, a total of 2,969 studies were identified from the databases, as mentioned above, within the last 10 years. Results were grouped in MS Excel to remove duplicates which were 40 in number. Out of the remaining papers, 2,839 studies were excluded based on irrelevant titles and abstracts. There were 90 studies left, which were thoroughly screened as full-text papers. Out of those, 62 were excluded. Quality assessment was then performed on the remaining 28 studies using quality assessment tools such as A Measurement Tool to Assess Systematic Reviews (AMSTAR 2) (for systematic reviews and meta-analyses), Scale for the Assessment of Narrative Review Articles (SANRA) (for narrative review articles), Appraisal Tool for Cross-Sectional Studies (AXIS) tool (for cross-sectional studies), and Newcastle-Ottawa Scale (for case-control and cohort studies). A total of 17 studies that scored ≥70% on quality assessment were included in this review. An overview of the screening process is presented in the PRISMA chart, as shown in Figure 1.

The studies included in this systematic review are summarized in Table 2.

**Table 2:** Characteristics of studies included in this systematic review. SANRA: Scale for the Assessment of Narrative Review Articles; AMSTAR: Assessment of Multiple Systematic Reviews; AXIS tool: Appraisal Tool for Cross-Sectional Studies

Author	Publication year	Report type	Quality assessment tool used	Score
Li et al. [3]	2022	Review article	SANRA	9
Wan and El-Nezani [18]	2018	Review article	SANRA	9
Li et al. [19]	2022	Review article	SANRA	9
Yu and Schwabe [20]	2017	Review article	SANRA	10
Huang et al. [21]	2022	Systematic review and meta-analysis	AMSTAR 2	12
Ponziani et al. [22]	2019	Cross-sectional study	AXIS tool	15
Zhang et al. [23]	2021	Cross-sectional study	AXIS tool	14
Ren et al. [24]	2019	Cross-sectional study	AXIS tool	14
Zheng et al. [25]	2020	Cross-sectional study	AXIS tool	15
Yan et al. [26]	2023	Cross-sectional study	AXIS tool	14
Temraz et al. [27]	2021	Review article	SANRA	10
Huang et al. [28]	2020	Cross-sectional study	AXIS tool	14
Piñero et al. [29]	2019	Case-control study	Newcastle-Ottawa scale	7
Sharpton et al. [30]	2019	Systematic review and meta-analysis	AMSTAR 2	12
Hariri et al. [31]	2023	Cohort study	Newcastle-Ottawa scale	7
Pohl et al. [32]	2022	Systematic review	AMSTAR 2	12
Wang et al. [33]	2022	Cohort study	Newcastle-Ottawa scale	7

Discussion

This section will focus on how the gut and liver are closely related and the role of the gut microbiome in the development, diagnosis, and treatment of HCC.

Gut-Liver Axis

The gastrointestinal tract (GIT) and liver are closely related to each other through the portal vein, the main source of blood supply to the liver. The liver regulates the function of the intestine by secreting bile acids into the intestine, while the portal vein brings the absorbed nutrients and products of gut flora, such as LPS, from the GIT to the liver [18]. The gut-liver axis is vital in developing liver diseases such as non-alcoholic fatty liver disease (NAFLD), cirrhosis, and HCC.

The intestinal mucosal barrier is a dynamic structure that prevents the entry of harmful gut flora and its metabolites into the blood. It has physical, chemical, and biological components consisting of intestinal mucosal epithelium, Paneth cells, mucosal-associated lymphoid tissue, and goblet cells [19]. Thus, the altered intestinal mucosal barrier affects the gut-liver axis. The intestinal mucosal barrier and gut flora regulate each other. The intestinal mucosal barrier produces antibacterial substances and scavenges gut flora. On the other hand, gut flora affects the growth of the intestinal mucosal barrier and epithelial cells and thus prevents the growth of harmful microbes such as *Clostridium difficile* [20].

About 95% of bile acids are reabsorbed from the distal ileum and brought to the liver by the portal vein through a process known as enterohepatic circulation. Farnesoid X receptor (FXR) is the main bile acid receptor present in the liver and ileum and regulates bile acid synthesis and secretion. The gut microbiome regulates bile acid synthesis in the liver by regulating the expression of FXR [3]. Therefore, the gut microbiome regulates secondary bile acid production in the gut and bile acid synthesis in the liver, which then regulates gut flora composition.

Role of Gut Microbiome in Hepatocellular Carcinoma Development

Gut microbiome dysbiosis is usually seen in patients with cirrhosis and HCC, resulting in increased pathologic bacteria and decreased beneficial bacteria. A meta-analysis by Huang et al. by combining 17 studies revealed an increased number of *Enterobacter *and* Enterococcus* and decreased number of *Lactobacillus *and *Bifidobacterium* in the gut of cirrhotic patients [21].

In a study done by Ponziani et al., which involved three groups, namely, NAFLD-related cirrhosis and HCC, NAFLD-related cirrhosis without HCC, and healthy controls, showed increased levels of *Lactobacillus*,* Haemophilus*,* Enterococcus*, and* Klebsiella* in the gut of cirrhotic patients compared to healthy people and decreased levels of *Akkermansia *and* Methanobrevibacter* compared to healthy people [22]. The study also revealed that patients with HCC had increased *Bacteroides*,* Enterococcus*,and* Ruminococcaceae* levels and reduced *Bifidobacterium *levels in their gut compared to patients with cirrhosis but without HCC.

A study by Zhang et al. involving HCC patients who were divided into three groups based on Barcelona Clinic Liver Cancer Staging showed increased levels of *Enterococcus *and* Enterobacteriaceae *and decreased levels of *Actinobacteria *and* Bifidobacterium* in the gut with increased severity of HCC [23].

A study by Ren et al. involving HCC patients, cirrhosis patients, and healthy individuals showed decreased levels of butyrate-producing bacteria such as *Ruminococcus*,* Oscillibacter*,and* Faecalibacterium* and increased levels of LPS-producing bacteria such as *Klebsiella *and* Haemophilus* in the gut of HCC patients compared to other groups [24]. The study also revealed decreased gut microbiome diversity in cirrhosis patients compared to other groups.

A study by Zheng et al. involving patients with hepatitis, cirrhosis, cirrhosis-induced HCC, non-cirrhosis-induced HCC, and healthy individuals showed increased levels of Bacteroidetes and *Fusobacteria *and microbiome diversity in the gut of HCC patients compared to other groups [25]. In contrast, gut microbiome diversity was decreased in cirrhosis patients compared to other groups, findings which were similar to the study done by Ren et al. [24]. Moreover, no significant difference in gut microbiome composition was seen in HCC patients with different etiologies.

A study done by Yan et al. involving patients with HBV-cirrhosis, HBV-HCC, and healthy people showed increased levels of proinflammatory bacteria such as *Proteus*,* Klebsiella*,and* Streptococcus* and reduced levels of butyrate-producing bacteria and beneficial bacteria such as *Bacteroides *and*Firmicutes *in the gut of HBV-cirrhosis and HBV-HCC patients compared to healthy patients [26]. This study also revealed that gut microbiome diversity decreased in HBV-HCC patients, which contradicts the findings of the study done by Ren et al. [24], which may be due to differences in sample size, data analysis methods, and 16S rRNA sequencing. Another study finding was that *Lactobacillus* is increased in the gut of HBV-HCC patients which contradicts other studies and may be due to a more severe cancer-related inflammatory response.

Gut microbiome dysbiosis promotes HCC development by increasing gut mucosal barrier permeability and by triggering liver inflammation [27]. Dysbiosis results in increased production of secondary bile acids from bile acids causing increased activation of hepatic NKT, increased expression of toll-like receptor (TLR) 2 by liver stellate cells, and activation of mammalian target of rapamycin in hepatocytes. Increased gut permeability results in increased entry of LPS, a cell wall component of gram-negative gut flora, to reach the Liver, which activates TLR4, thus promoting liver carcinogenesis and liver fibrosis by triggering chronic inflammation.

Gut dysbiosis is associated with decreased levels of short-chain fatty acid (SCFA)-producing bacteria that degrade dietary fibers into SCFA, mainly acetate and butyrate [23]. Enterocytes use SCFA as an energy source and help maintain intestinal tight junction. Thus, reduced SCFA-producing bacteria can increase intestinal mucosal barrier permeability. The study also showed increased translocation of the gut microbiome with HCC progression characterized by increased levels of LPS-binding protein and LPS. Another study finding was elevated IL-6 and IL-8, which play a role in inflammation by activating neutrophils.

In different studies, gut microbial composition in HCC patients was different, possibly due to different dietary habits, causes of HCC, and topographical habitats. Moreover, the difference in gut microbiome profile between HCC and cirrhosis-only patients was smaller than that between healthy and cirrhosis patients. Thus, the role of the gut microbiome in the pathogenesis of HCC may be mainly due to cirrhosis-related changes rather than HCC-related changes.

Role of Gut Microbiome in Diagnosis, Prevention, and Treatment of Hepatocellular Carcinoma

A study by Ren et al. validated the diagnostic potential of a particular set of gut microbiomes as markers for early and late HCC [24]. A study by Ren et al. involving HBV-HCC patients (who were divided into small HCC and non-small HCC groups depending on tumor load) and healthy individuals showed that levels of *Bacteroides*, *Lachnospiracea incertae sedis*,and* Clostridium XIVa* were higher in the gut of non-small-cell HCC group (higher tumor load) and could separate the non-small-cell liver cancer group from small-cell HCC group (lower tumor load), indicating the use of gut microbiome in both the diagnosis and prognosis of HCC [28]. A study by Zheng et al. found that a few microbial genera were related to HCC tumor size, of which three (*Enterococcus*,* Limnobacter*,and* Phyllobacterium*) could be used as markers for the early diagnosis of HCC [25]. A study done by Pinero et al. involving cirrhotic patients with and without HCC showed increased levels of genus *Odoribacter* and *Butyricimonas* but decreased levels of genus *Dorea* in the gut of HCC patients compared to those without HCC and identified them as potential biomarkers of HCC [29]. As the difference in the gut microbiome profile between HCC patients and cirrhosis-only patients is smaller compared to the difference between healthy people and cirrhosis patients, HCC diagnostic tests based on the gut microbiome composition may have more power in diagnosing cirrhosis compared to HCC.

As mentioned earlier, gut microbiome dysbiosis plays a vital role in HCC development and progression; thus, manipulating the gut microbiome can prevent or treat HCC. Probiotics are microbes with health benefits, such as *Lactobacillus*,* Bifidobacterium*, and* Clostridium XIVa*, and are found to reduce the risk of HCC by promoting the growth of beneficial bacteria, which reduce inflammation by producing anti-inflammatory metabolites and reducing the expression of TLR [27]. Probiotics also increase bacteria that produce SCFA, such as *Butyricimonas* and *Prevotella*, and prevent HBV and HCV infections, thereby reducing HCC progression [18]. In NAFLD patients, probiotics have been shown to reduce aspartate aminotransferase, alanine aminotransferase, and total cholesterol and reduce liver inflammation by lowering tumor necrosis factor-alpha levels [27]. Probiotics can transform dietary fibers into metabolites having tumor-suppressive activity. A systematic review by Sharpton et al. involving 21 studies showed that the use of probiotics and synbiotics reduced liver inflammation, fibrosis, and steatosis in NAFLD patients, indicating a possible protective effect of probiotics and synbiotics in the prevention of HCC development [30]. A study by Hariri et al. showed that dietary fibers act as prebiotics and reduce liver inflammation by decreasing intestinal microbial dysbiosis, decreasing intestinal epithelial permeability, and thereby preventing the development of cirrhosis and HCC and mortality in cirrhotic patients [31].

Fecal microbiota transplantation (FMT) is a process by which fecal microbes are transplanted from a healthy person into the GIT of another person to alter gut microbiota. FMT can prevent HCC development by altering gut microbiota, resulting in decreased production of proinflammatory and cytotoxic metabolites and reduced gut microbiome dysbiosis [19]. FMT has shown promising results in inflammatory bowel disease and *C. difficile* patients, but its role in HCC remains unclear.

Antibiotics can stop HCC progression in chronic liver disease patients by decreasing the number of bacteria that can translocate and produce harmful metabolites [20]. Antibiotics have shown positive effects in animal studies, but their role in humans remains unclear. Currently, norfloxacin and rifaximin have demonstrated positive outcomes in chronic liver disease patients. A systematic review by Pohl et al., which included 17 studies, showed that increasing SCFA in the gut resulted in decreased gut permeability and liver inflammation [32]. However, more research is needed to find effective methods of increasing intestinal SCFA levels.

Effect of Altered Gut Microbiome on Cancer Immunotherapy

In HCC patients who responded to anti-programmed cell death protein 1 (PD-1) immunotherapy, Bacteroidetes remained the most common gut bacteria with increased levels of *Ruminococcacae* and *Akkermansia* *muciniphila* after treatment which is in contrast to patients who did not respond to anti-PD-1 therapy and had increasing levels of *Proteobacteria *after treatment, which became the most common taxon by the 12th week after treatment [3,27]. This indicates that gut microbiota can affect anti-PD-1 immunotherapy in HCC patients. A study by Wang et al. indicated that the use of antibiotics within 30 days before or after starting immunotherapy in liver cancer patients had no unfavorable effect on the outcome of immunotherapy which is unlike other cancers where adverse effects are noted on immunotherapy outcomes possibly due to difference in the composition of immune microbiota between liver cancer and other cancers [33].

Limitations

This systematic review has some limitations. Only free full-text articles published in the last 10 years and available across various databases were used in this review. Publications in languages other than English were not included in this review. Furthermore, individual studies included in this review had smaller sample sizes, but the cumulative sample size is sufficient to draw conclusions.

## Conclusions

This systematic review provides information regarding the gut-liver axis and how gut microbiome and liver diseases such as HCC affect each other. It was demonstrated that gut microbiome dysbiosis is seen in liver diseases such as HCC and how gut microbiome dysbiosis plays a role in HCC development, its diagnosis, as well as its prevention and treatment. It was also demonstrated that gut microbiome dysbiosis affects the response to HCC immunotherapy and can act as a prognostic marker. Suggestions for future research include more human trials with a larger number of subjects belonging to wider demography, as well as further research on the use of gut microbiome for the early detection of HCC, the use of antibiotics and probiotics in HCC treatment, and how gut microbiome dysbiosis affects the response to current HCC immunotherapy.
